# Urinary Tract Infections in Kidney Transplant Patients Due to *Escherichia coli* and *Klebsiella pneumoniae*-Producing Extended-Spectrum β-Lactamases: Risk Factors and Molecular Epidemiology

**DOI:** 10.1371/journal.pone.0134737

**Published:** 2015-08-03

**Authors:** Maria José Espinar, Isabel M. Miranda, Sofia Costa-de-Oliveira, Rita Rocha, Acácio G. Rodrigues, Cidália Pina-Vaz

**Affiliations:** 1 Department of Microbiology, Faculty of Medicine, University of Porto, Porto, Portugal; 2 Clinical Pathology Department, Hospital São João, Porto, Portugal; 3 CINTESIS-Center for Health Technology and Services Research, Faculty of Medicine, University of Porto, Porto, Portugal; California Department of Public Health, UNITED STATES

## Abstract

Urinary tract infection (UTI) is a common complication after kidney transplantation, often associated to graft loss and increased healthcare costs. Kidney transplant patients (KTPs) are particularly susceptible to infection by *Enterobacteriaceae*-producing extended-spectrum β-lactamases (ESBLs). A retrospective case-control study was conducted to identify independent risk factors for ESBL-producing *Escherichia coli* and *Klebsiella pneumoniae* in non-hospitalized KTPs with UTI. Forty-nine patients suffering from UTI by ESBL-producing bacteria (ESBL-P) as case group and the same number of patients with UTI by ESBL negative (ESBL-N) as control-group were compared. Clinical data, renal function parameters during UTI episodes, UTI recurrence and relapsing rate, as well as risk factors for recurrence, molecular characterization of isolates and the respective antimicrobial susceptibility profile were evaluated. Diabetes mellitus (*p* <0.007), previous antibiotic prophylaxis (*p*=0.017) or therapy (*p*<0.001), previous UTI (*p*=0.01), relapsing infection (*p*=0.019) and patients with delayed graft function after transplant (*p*=0.001) represented risk factors for infection by ESBL positive *Enterobacteriaceae* in KTPs. Interestingly, the period of time between data of transplantation and data of UTI was shorter in case of ESBL-P case-group (28.8 months) compared with ESBL-N control-group (50.9 months). ESBL-producing bacteria exhibited higher resistance to fluoroquinolones (*p*=0.002), trimethoprim-sulfamethoxazole (*p*<0.001) and gentamicin (*p*<0.001). Molecular analysis showed that *bla*_CTX-M_ was the most common ESBL encoding gene (65.3%), although in 55.1% of the cases more than one ESBL gene was found. In 29.4% of *K*. *pneumoniae* isolates, three *bla*-genes (*bla*_CTX-M_-*bla*_TEM_-*bla*_SHV_) were simultaneously detected. Low estimated glomerular filtration rate (*p*=0.009) was found to be risk factor for UTI recurrence. Over 60% of recurrent UTI episodes were caused by genetically similar strains. UTI by ESBL-producing *Enterobacteriaceae* in KTPs represent an important clinical challenge regarding not only hospitalized patients but also concerning outpatients.

## Introduction

Urinary tract infections (UTI) affect 5 to 36% of kidney transplant patients (KTPs), being the main infectious complication among such patient population [1–4], and one of the most common causes of graft loss and mortality [5,6]. Interestingly, we found a high incidence of extended-spectrum β-lactamases (ESBL) producing bacteria (49%) among KTPs at a large University hospital in Portugal, Centro Hospitalar São João (unpublished data). Usually, infections caused by ESBL-producing bacteria are associated with increased morbidity and mortality which entails enhanced healthcare costs [5,7]. Therefore, UTI caused by ESBL-producing bacteria in KTPs is of extremely clinical relevance.

ESBLs are a large, rapidly evolving group of plasmid-enzymes that confer resistance to penicillins, first-, second-, and third generation cephalosporins, and aztreonam. They are inhibited by beta-lactamase inhibitors such as clavulanic acid [8–10]. During recent years, antibacterial therapy became increasingly more complex. ESBL positive isolates are often associated with multidrug resistance (MDR) especially to fluoroquinolones, aminoglycosides and sulfonamides [11,12]. Insertion sequences, integrons and transposons promiscuously transferred between bacteria played a crucial role in global dissemination of the most common ESBL genes, namely *bla*_CTX-M_ [13]. The prevalence of ESBL-producing organisms increased dramatically in the last decade in particular as an etiological agent in community-acquired infections, health care settings, nursing homes, and even veterinary settings [14–17]. There are several recognized risk factors associated with UTI in KTPs [18–20], however very few studies evaluated selective risk factors for infection by ESBL-producing bacteria in such patients, as well as, its respective molecular epidemiology and antimicrobial susceptibility profiles.

The aim of this study was to identify risk factors, the susceptibility profile and recurrence associated to UTI caused by ESBL-producing *E*. *coli* and *K*. *pneumonia*e comparatively to the respective non ESBL-producing counterparts. ESBLs encoding genes were identified. *E*. *coli* and *K*. *pneumoniae* isolates sequentially recovered from each patient were genotyped to assess about re-infection or bacterial persistence.

## Materials and Methods

### Study Design

This study was approved by Comissão de Etica para a Saúde (CES) do Centro Hospitalar de São João (Approval reference number: 256/13), signed by Prof. Manuel Pestana (Director of Nephrology Department). Patient records/information was anonymized and de-identified prior to analysis.

A retrospective case-control study enrolling 49 outpatients with UTI by *E*. *coli* and *K*. *pneumoniae* ESBL-producing bacteria (ESBL-P), as case-group, and 49 outpatients with UTI by the same species but ESBL negative (ESBL-N), as a control-group, was performed. All patients were kidney transplant patients (KTPs) from Centro Hospitalar São João, Porto (Portugal) with documented UTI between January 2012 and August 2012.

### Variables

Demographic data (age, gender) and clinical variables such as delayed graft function, the presence of urinary tract obstruction, bacteremia, fungemia, previous urinary tract infection, creatinine blood level (reference range 0.6–1.1 mg/dL), and the percentage of patients with low estimated glomerular filtration rate (eGFR) during UTI (<60 mL/min/1.72m^2^) was registered [21]; eGFR was calculated using a MDRD (Modification of Diet in Renal Disease) study equation [22] during UTI. Additional data such as leukocyturia, erithrocyturia, urinary nitrites, immunosuppression therapeutic regimen, antibiotic prophylaxis, antibiotic therapy (comprising the two previous months) was recorded. Patient comorbidities such as hypertension, coronary disease and diabetes mellitus (either preexistent or post-transplantation), Cytomegalovirus (CMV) infection, percentage of graft loss and mortality, along a 3 months period, were also registered. It was calculated, in months, the period between post-transplant data and this UTI episode. A comparison of antimicrobial susceptibility profile between case-group (ESBL-P) and control-group isolates (ESBL-N), as well as, between successive isolates from the same patient was also performed. Recurrent UTI was defined as two episodes separated at least 1 month, during the following three months. Relapsing UTI was defined as two episodes caused by the same species with an identical antibiogram after appropriate treatment [23].The percentage and risk factors for recurrence were calculated for both groups.

### Phenotype characterization and Molecular analysis of clinical isolates

Eighty-two isolates of ESBL-producing *E*. *coli* (n = 50) and *K*. *pneumoniae* (n = 32) were recovered from 49 KTPs (case-group): 25 patients yielded each a single isolate, 16 patients two isolates each, 7 patients three isolates each, one single patient yielding four isolates. Forty-nine ESBL negative isolates (32 *E*. *coli* and 17 *K*. *pneumoniae*) were recovered from the 49 patients of control group. All isolates were analyzed by Vitek2 System (BioMérieux, Marcy L´Etoile, France) using GN card for identification and the AST-60 for antimicrobial susceptibility profile. ESBL presence was confirmed by disc diffusion method, according to the recommendations of Clinical Laboratory Standard Institute [24]; a double-disk synergy test with cefotaxime (30 μg) and ceftazidime (30 μg) alone and in combination with clavulanic acid (10 μg) (Oxoid, Hampshhire, U.K.) was performed in Mueller-Hinton agar. Quality control of susceptibility tests was carried out using *Escherichia coli* ATCC 25922 and *Klebsiella pneumoniae* ATCC700603 type strains, as recommended by CLSI protocol [24] and plasmid DNA was extracted using the NZY Miniprep Kit (NZYTech, Lisbon, Portugal). Genomic DNA was extracted according the “boiling method”. DNA quantification and quality was assessed in a Nanodrop 2000c equipment (Thermo Fisher Scientific, CA, U.S.). Plasmid and genomic DNA were visualized in a Molecular Imager ChemiDoc XRS (BioRad, CA, U.S.) after electrophoresis (80 V, 1 hour) in a 0.8% agarose gel.

The most frequently ESBL genes (*bla*_CTX-M,_ *bla*_TEM_, and *bla*_SHV_) were screened by PCR multiplex, as previously described [25]. The primers used were: *bla*_SHV_: 5´-ATCCACTATCGCCAGCAGG-3´ and 5´-TCATTCAGTTCCGTTTCCCAG-3´; *bla*_TEM_: 5´-GAGTATTCAACATTTCCGTGTC-3´ and 5´-GGGCGAAAACTCTCAAGGATC-3´; *bla*_CTX-M_: 5´-GTTGTTAGGAAGTGTGCCGC-3´ and 5´-GCCCGAGGTGAAGTGGTATC-3´. Primers amplified the internal fragments of ESBLs genes possessing different sizes. Multiplex PCR was performed in a 25 μl reaction mixture containing 1x Dream Taq buffer (Fermentas), 2.5 mM MgCl_2_, 10 mM dNTPs, 2.5–40 pmol of specific-group primers, 50–150 ng plasmid DNA and 1 U of Dream Taq Polymerase (Fermentas). Amplification reactions were carried out in Mastercycler *Realplex* ^2^ (Eppendorf) under the following conditions: initial denaturation at 95°C for 2 min, followed by 30 cycles of denaturation (95°C for 30 sec), annealing (59°C for 30 sec), extension (72° for 30 sec) and a final extension step (72°C for 10 min). PCR products were run at 80 V, during 1 hour in a 2.5% agarose gel containing 0.002% Ethidium Bromide (AppliChem, Darmstadt, Germany). ESBLs bacteria exhibiting well-characterized ESBL genes kindly gift by Dr. Rafael Cantón (Servicio de Microbiología, Hospital Universitario Ramón y Cajal) were used as positive controls.

Sequential isolates from case-group (ESBL-P)- 2 or more isolates (24 patients)—were genotyped by RAPD. Distinct primers were used for *E*. *coli* [26] (GTGATCGCAG) and *K*. *pneumoniae* [27] (ACGTATCTGC) isolates. Each reaction contained: 1x DreamTaq buffer (Fermentas, Thermo Fisher Scientific, CA, U.S.), 0.2 mM of dNTPs, 0.5 pmol of primer, 14 mM of dimethylsulfoxide (Merck, NJ, U.S.), 10 ng of genomic DNA and 0.6 U of DreamTaq polymerase (Fermentas). PCR protocol was performed in a Mastercycler *Realplex* ^2^ thermocycler (Eppendorf, Wesseling-Berzdorf, Germany), and involved the following steps: initial denaturation at 94°C for 1 min, followed by 45 cycles of denaturation (94°C at 1 min), annealing (34°C at 1 min), extension (72°C at 2 min), and a final extension step (72°C at 10 min). PCR products were separated by electrophoresis (60 V for 3 hours) in a 2.5% agarose gel. DNA fingerprints were compared by visual inspection with the Molecular Imager ChemiDoc XRS (BioRad). Clonality was assessed by visual inspection of the different fragments obtained, in a range of 2000–200 bp for *E*. *coli* and 3000–250 bp for *K*. *pneumoniae*. According to *Wong et al*. [28], isolates displaying identical RAPD profiles or showing 3 minor band variations were considered the same strain.

### Statistical analysis

Statistical analysis was performed using IBM SPSS statistical package (Chicago, IL) version 20. Continuous variables were summarized as mean value and standard deviation (SD). Categorical variables, summarized as percentages, were compared using Chi-square test (or Fishers ‘exact test whenever was necessary) and Student´s *t*- test. Multivariable logistic regression analysis, using a likelihood ratio-based backward exclusion method, was used to evaluate independent risk factors associated with ESBL positive UTI. A two-sided *p*<0.05 value was considered to be statistically significant.

## Results

### Clinical data and risk factors

Patient clinical data, including demographic characteristics, co-morbidities, clinical laboratory data, immunosuppressive therapeutic regimen, previous UTI and antibiotic exposure are summarized in Table 1. The number of patients with diabetes mellitus, with low eGFR, under antibiotic (prophylaxis or therapy), with history of previous UTI, relapsing UTI, and delayed graft function were significantly higher in the ESBL-P group; the mean period of time between data of transplantation and UTI episode was shorter in case of ESBL-P case group (Table 1).

**Table 1:** Comparative analysis of clinical and laboratorial data from kidney transplant patients yielding extended spectrum β-lactamase positive (ESBL-P) and extended spectrum β-lactamase negative (ESBL-N) *Escherichia coli* and *Klebsiella pneumoniae* isolated from urine. Predicted risk factors were obtained after univariate and multivariate (logistic regression) analyses.

	ESBL Positive Case-group	ESBL Negative Control-group	*p*	OR*	OR*adjusted	CI 95%**	*p*
Number of patients	49	49					
Male gender	17 (34.7%)	18 (36.7%)					
Mean age (SD)	53.8 (14.8)	51.6 (13.5)					
Comorbilities							
Diabetes mellitus	20 (40.8%)	8 (16.3%)	0.007	3.53	6.81	1.75–26.39	0.006
Hypertension	32 (65.3%)	32 (65.3%)	NS	-	-	-	-
Coronary disease	11 (20.4%)	11 (24.5%)	NS	-	-	-	-
Cytomegalovirus Infection	2 (4.1%)	2 (4.1%)	NS	-	-	-	-
Urinary Nitritus	6 (12.5%)	13 (26.5%)	NS	-	-	-	-
Eritrocyturia (mean value)	120.6/μL	358,7/μL					
Leucocyturia (mean value)	333.0/μL	243.2/μL					
Low eGFR during UTI^##^	39 (79.6%)	28 (57.1%)	0.017	1.4	-	-	-
Immunosupression Regimen							
Tacrolimus	26 (53%)	23 (46.9%)	NS	-	-	-	-
Ciclosporine	18 (36.7%)	22 (44.9%)	NS	-	-	-	-
Everolimus	2 (4.1%)	4 (8.2%)	NS	-	-	-	-
Antibiotic prophylaxis	24 (49%)	14 (28.6%)	0.038	2.40	3.73	1.09–12.78	0.036
Previous antibiotic therapy	34 (69.4%)	13 (26.5%)	<0.001	6.27	15.8	4.28–58.5	0.000
Previous UTI	39 (79.6%)	28 (57.1%)	0.01	4.92	7.73	1.90–31.36	0.004
Recurrence UTI	23 (46.9%)	20 (40.8%)	NS	-	-	-	-
Relapsing UTI	22 (44.9%)	11 (22.4%)	0.019	2.81	3.42	1.00–11.66	0.049
Delayed graft function	27 (62.8%)	11 (27.5%)	0.001	4.44	4.1	1.25–13.61	0.020
Mortality	2 (4%)	0 (0%)	NS	-	-	-	-
Mean period of time between data of transplantation and UTI (months)	28.8	50.9	0.011	-	-	-	-

UTI = Urinary tract infection; NS = not significant;

^##^Low eGFR = <60 ml/min/1.73m^2^;

^¶^Mortality in the next 3 months after infection;

*Odds Ratio;

** Confident interval at 95%;

^∞^15 patients without data.

Regarding immunosuppression, all patients showed adequate therapeutic levels; mean values were: 8.1 ng/mL for tacrolimus, 190.5 ng/mL for cyclosporine and 6.28 ng/mL for everolimus. Patient death occurred solely among ESBL-P group, during the 3 months after infection (Table 1). No bacteremia or fungemia, urinary tract obstruction or graft loss was found among both patients groups. UTI recurrence frequency, up to 46.9%, was similar in both groups (Table 1). The analysis of the risk factors associated to UTI by ESBL-positive bacteria revealed that diabetes mellitus, antibiotic prophylaxis, antibiotic therapy during the previous 2 months, previous UTI, relapsing UTI and delayed graft function contributed independently (Table 1). Prophylactic antibiotic therapy was more often used in ESBL-P group; it consisted of oral trimethoprim/sulfamethoxazole during at least 12 months. Antibiotic therapy, during the two months previous to UTI, involved mainly trimethoprim-sulfamethoxazole (48.9%), ceftriaxone (21.3%) and piperaciline-tazobactam (17.1%) therapeutics. During UTI episode, amoxicillin associated to clavulanic acid was frequently administrated or alternatively in case of an ESBL positive isolate, a carbapenem for a 10 day period.

### Antimicrobial susceptibility profile

The comparative analysis of the antimicrobial susceptibility profiles showed statistically significant differences between isolates from ESBL-P and ESBL-N groups, in what concerns fluoroquinolones, trimethoprim-sulfamethoxazole and aminoglycosides. No significantly differences were seen regarding *K*. *pneumoniae* susceptibility to amikacin between both study groups (Table 2). No resistance to carbapenems was found in both groups.

**Table 2:** Comparative analysis of the distribution of antimicrobial resistant extended spectrum β-lactamase positive (ESBL-P) and extended spectrum β-lactamase negative (ESBL-N) isolates (n/%) recovered from urinary tract infection of kidney transplant patients.

	ESBL Positive Case-group	ESBL NegativeControl-group	*p*
*E*. *coli-* Resistance to			
Ciprofloxacin	16 / 48.5%	5 / 15.6%	0.004
Levofloxacin	16 / 48.5%	5 / 15.6%	0.002
Norfloxacin	12 / 36.4%	6 / 18.8%	<0.001
Cotrimoxazol	26 / 78.8%	19 / 59.4%	0.036
Gentamicin	8 / 24.2%	2 / 6.2%	0.044
Amikacin	5 / 15.2%	0 / 0%	0.022
*K*. *pneumoniae-*Resistance to			
Ciprofloxacin	6 / 37.5%	4 / 22.5%	0.028
Levofloxacin	6 / 37.5%	0 / 0%	0.020
Norfloxacin	6 / 37.5%	3 / 17.6%	0.004
Cotrimoxazol	15 / 93.8%	9 / 47.1%	0.002
Gentamicin	9 / 56.2%	1 / 5.9%	0.002
Amikacin	4 / 25.0%	3 / 17.6%	NS
Total strains- Resistance to			
Ciprofloxacin	22 / 44.9%	9 / 18.4%	0.002
Levofloxacin	22 / 44.9%	5 / 10.2%	<0.001
Norfloxacin	18 / 36.7%	9 / 18.4%	<0.001
Cotrimoxazol	41 / 83.7%	34 / 55.1%	<0.001
Gentamicin	16 / 32.7%	2 / 4.1%	0.002
Amikacin	2 / 4.1%	0 / 0%	NS

NS = not significant

### Molecular characterization of ESBL genes

ESBL genes (*bla*_CTX-M,_ *bla*_SHV_, and *bla*_TEM_ group) were detected by PCR multiplex in isolates from ESBL-P group (Table 3). Interestingly, sequential isolates recovered from the same patient express the same ESBL genes. The gene most frequently expressed was *bla*_CTX-M_ (65.3%), either alone (18.4%) or in combination with another ESBL gene (46.9%), followed by *bla*_TEM_ (61.2%) and *bla*_SHV_ (40.8%). More than half of the case- group express more than one ESBL gene, being the association *bla*_CTX-M_ *- bla*_TEM_ the most frequent (28.5%), particularly among *E*. *coli* strains (37.5%). Among *K*. *pneumoniae* isolates, *bla*_CTX-M_-*bla*_TEM_-*bla*_SHV_ (29.4%) was the most frequent association, followed by *bla*_CTX-M_-*bla*_SHV_ (23.5%). Strains expressing a single ESBL gene, *bla*_CTX-M_ (25%) and *bla*_TEM_ (18.8%) corresponded most often to *E*. *coli*, while *bla*_SHV_ was more common among *K*. *pneumoniae* isolates (17.6%) (Table 3). No correlation was found between ESBL genotype and parameters such as: antimicrobial resistance, clinical variables such as diabetes mellitus, previous antibiotic prophylaxis/treatment and UTI recurrence.

**Table 3:** Extended spectrum β-lactamase (ESBL) genotypes of *Escherichia coli* and *Klebsiella pneumoniae* isolates from urine of kidney transplant patients.

ESBL genes	*E*.*coli*n = 32 (%)	*K*.*pneumoniae*n = 17 (%)	Total strainsn = 49 (%)
**Single ESBL gene**			
*bla*_CTX-M_	8 (25%)	1 (5.9%)	9 (18.4%)
*bla*_TEM_	6 (18.8%)	3 (17.6%)	9 (18.4%)
*bla*_SHV_	3 (9.4%)	3 (17.6%)	6 (12.4%)
			24 (48.9%)
**Two or more ESBL genes**			
*bla*_CTX-M_ *+ bla*_TEM_	12 (37.5%)	2 (11.8%)	14 (28.5%)
*bla*_CTX-M_ *+ bla*_SHV_	0 (0%)	4 (23.5%)	4 (8.2%)
*bla*_TEM_ *+ bla*_SHV_	2 (6.3%)	2 (11.8%)	4 (8.2%)
*bla*_CTX-M_ *+ bla*_SHV_ *+ bla*_TEM_	1 (3.1%)	5 (29.4%)	6 (12.2%)
			28 (57.1%)
**Overall genes**			
*bla*_CTX-M_	21 (65.6%)	11 (64.7%)	32 (65.3%)
*bla*_TEM_	21 (65.6%)	9 (52.9%)	30 (61.2%)
*bla*_SHV_	6 (18.7%)	14 (82.3%)	20 (40.8%)

### Recurrence of ESBL-P strains

**Fig 1:**
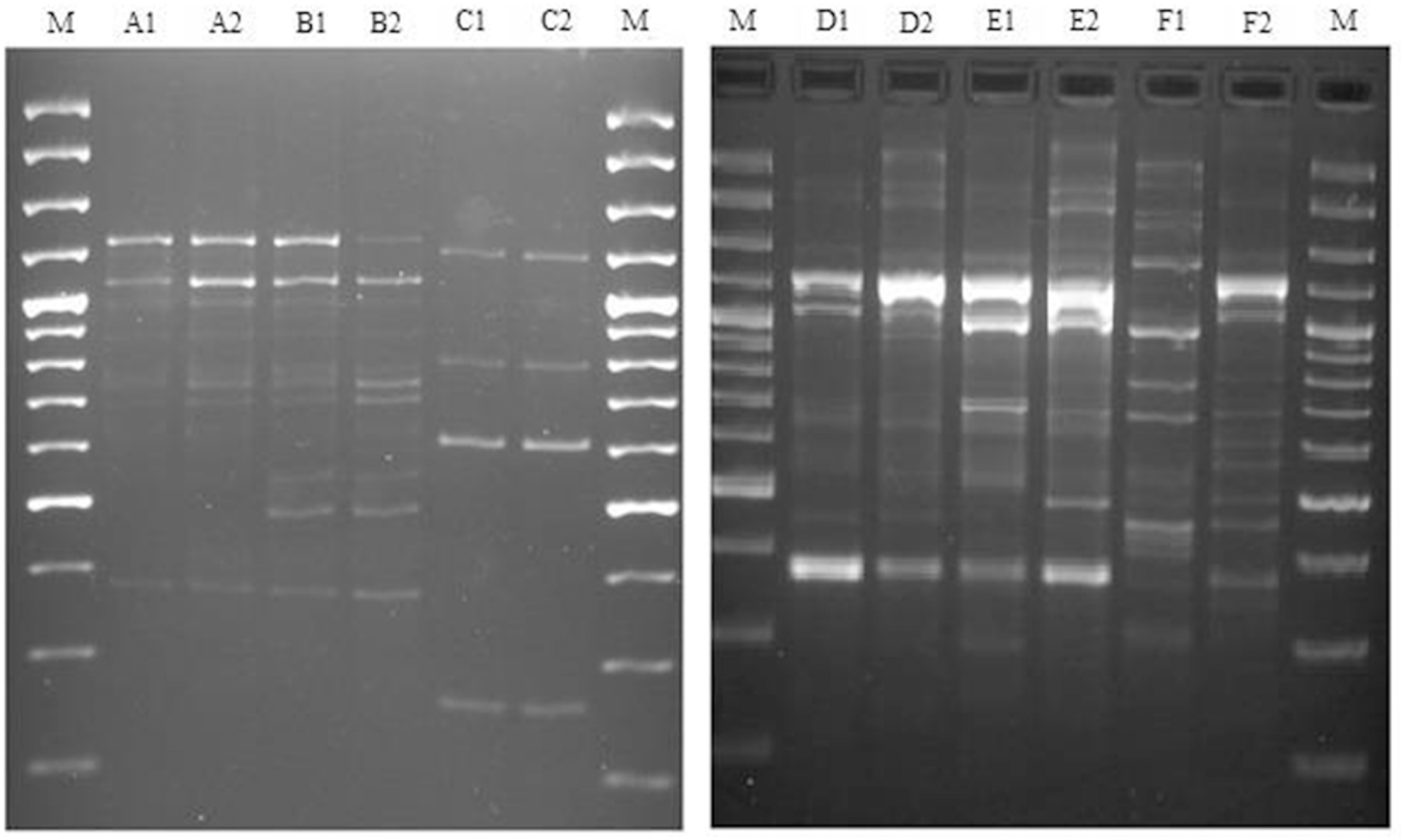
Recurrent UTIs are caused by clonal ESBL producing bacteria. Representative example of RAPD patterns of 6 strains of extended spectrum β-lactamase positive *Escherichia coli* (left panel; patients A, B, C) and 6 strains of extended spectrum β-lactamase positive *Klebsiella pneumoniae* (right panel; patients D, E, F); (1) represents strains recovered from the first urinary tract infection episode and (2) represents the second isolate recovered from the same patient. M- DNA Ladder 100 bp (Fermentas).

Although the level of recurrence found for both groups was similar (around 40%), the relapsing percentage was significantly higher on control-group (Table 1). The 48 ESBL-P isolates recovered from 24 patients with recurrent UTI (over 60%) showed similar antimicrobial susceptibility profiles (relapsing cases) and similar RAPD patterns (61.5% in the case of *E*. *coli* and 63.6% in the case of *K*. *pneumoniae*) (see representative examples in Fig 1). Conversely, isolates from 37.5% of the patients with recurrent UTI exhibited distinct RAPD patterns (see representative examples in Fig 1); such recurrent isolates exhibited invariably increased antimicrobial resistance, in particular to gentamicin and quinolones (data not shown). Comparative analysis of patients with and without UTI recurrence revealed that low eGFR was risk factor for recurrence (Table 4).

**Table 4:** Comparative analysis between clinical and laboratorial data of kidney transplant patients with urinary tract infection recurrence; molecular characterization of ESBL *Escherichia coli* and *Klebsiella pneumoniae* isolates.

	Patients with UTI recurrence	Patients without UTI recurrence	*p*	OR*	CI 95%**
Number of patients	43	55			
Diabetes mellitus	14 (32.6%)	14 (25.5%)	NS	-	-
Hypertension	31 (72.1%)	33 (60.0%)	NS	-	-
Coronary disease	10 (23.3%)	12 (21.8%)	NS	-	
Low eGFR during UTI^##^	35 (81.4%)	31 (56.4%)	0.009	0.295	0.116–0.752
Urinary Nitrites	9 (20.9%)	11(20.5%)	NS	-	-
Antibiotic prophylaxis	15 (34.9%)	23 (41.8%)	NS	-	-
Previous antibiotic therapy	22 (51.2%)	25 (46.5%)	NS	-	-
Previous UTI	30 (69.8%)	37 (67.3%)	NS	-	-
*bla* genes	23 (53.5%)	26 (47.3%)	NS	-	-

UTI = Urinary tract infection;

^##^Low eGFR = <60 ml/min/1.73m^2^;

*Odds Ratio;

** Confident interval at 95%

## Discussion

Kidney transplant recipients display many risk factors for UTI and are considered a particular vulnerable population to such infections [1]. Several studies have evaluated risk factors for UTI in community infections [11,18,29] and health care settings [20,30,31]. The high incidence of ESBL positive infections among KTPs with diabetes mellitus, as well as, in patients who received previous antimicrobial therapy were also reported by several authors [19,20]. Most of those studies report to hospitalized patients being the outpatients studies much more scarce.

Similar to Linares study [19] impairment of renal function, measured by delayed graft function, was risk factor for UTI due to ESBL-positive bacteria. The significant reduction of eGTF during UTI among KTPs [18] and high creatinine values [32] had already been reported by previous authors [18]. However, we demonstrated that such reduction is significantly higher among patients with UTI caused by ESBL-P bacteria probably due to the existence of acute kidney injury because of delayed graft function. For the first time, patients with low eGTF showed a risk factor for UTI recurrence, fact not found in Wu *et al*. study [29]. Remarkably, the period of time between data of transplant and the instauration of UTI episode by ESBL-P bacteria was shorter than ESBL-N group. This fact it could be due to delayed graft function which may will be predispose infection by bacteria. On other hand, antibiotic (prophylaxis or therapeutic) can induce the acquisition of antibiotic resistance genes predisponing to ESBL-P bacteria infection. PCR multiplex of the three most prevalent ESBLs genes (*bla*_CTX-M,_ *bla*_TEM_, and *bla*_SHV_) in *E*. *coli* and *K*. *pneumoniae* revealed that *bla*_CTX-M_ was the most common ESBL gene. A higher predominance of *bla*_CTX-M_ enzyme among ESBL positive *E*. *coli* was reported in Greece [33]. Conversely, extremely variable rates among different centres (1.2 to 49.5%) were described in Italy [34]. In Portugal, national surveys are not available. Nevertheless, studies from individual hospitals reflect a common spread of *bla*_CTX-M_ and *bla*_TEM_ [35,36]. Reports describing the co-production of different ESBLs by clinical isolates are increasing among European countries [37]. A high prevalence of *E*. *coli* and *K*. *pneumoniae* (57.3%) isolates exhibiting two or three genes was previously reported [38], similar to our study. Organisms producing *bla*_SHV_ and *bla*_TEM_ types of ESBLs have traditionally been responsible for serious health care related infections [39] while *bla*_CTX-M_ types have been mainly associated with community-onset UTI [40]. The epidemiology of ESBL-producing bacteria is becoming more complex and the limits between hospital and community settings are narrowing [15], being this fact particularly true in the case of KTPs. Such out-patients are frequently admitted to hospital. Thus, microorganism origin may not be obvious since patients can acquired an ESBL positive strain during the last hospital admission, and just after discharge a subsequent UTI can manifest. While other studies found a correlation between *bla*_CTX-M_-producing bacteria and resistance to fluoroquinolones [11] such correlation was not found in our study population. Regarding RAPD similar patterns corresponded to identical antimicrobial susceptibility profiles, while diferent RAPD patterns correspond to distinct profiles. Interestingly, recurrent isolates with different RAPD patterns showed invariably higher antimicrobial resistance, suggesting a complex, yet unveiled concomitance of multiple mechanism of resistance.

In conclusion, we have demonstrated that delayed graft function, diabetes mellitus, previous antibiotic exposure, antibiotic prophylaxis and relapsing UTI are independent risks factors for acquiring infections by ESBL-producing *E*. *coli* and *K*. *pneumoniae*. Molecular epidemiology showed that *bla*_CTX-M_ was the most common ESBL encoding gene, either alone or in association with other genes. Low eGFR and high blood creatinine are risk factors for UTI recurrence. The high co-resistance to other antibiotics (non-β-lactams) found from ESBL producing bacteria in UTI from KTPs, remains a serious clinical challenge.
